# Supplementary figures and images for: Mechanisms and potential immune tradeoffs of accelerated coral growth induced by microfragmentation
Source: PeerJ. 2022 Mar 29;10:e13158. doi: 10.7717/peerj.13158 (PMC8973463; doi:10.7717/peerj.13158)

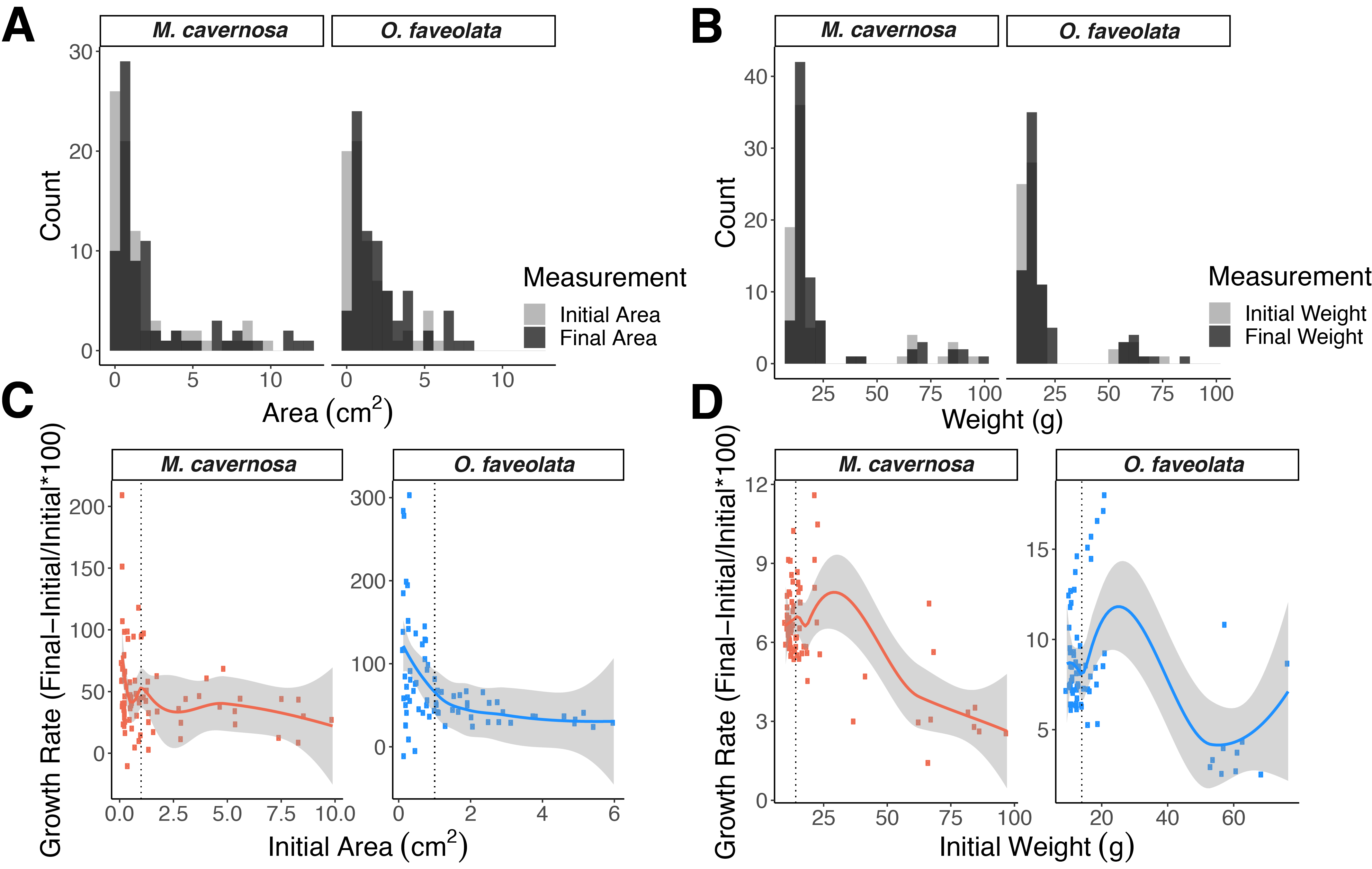

Supplement: Supplemental Information 1 — Histograms showing the range of initial (grey) and final (black) sizes for each coral species as area (A) and buoyant weight (B). Relationships between initial area (C) and initial buoyant weight (D) and percent increase. Points represent individual fragments. The solid line represents a locally weighted smoothing for the scatter plot with 95% confidence intervals shaded in grey. The vertical dotted lines indicate typical values associated with “microfragments”: initial areas of ~1 cm2 (C) or initial buoyant weights of ~14 g (D). [file peerj-10-13158-s001.png]

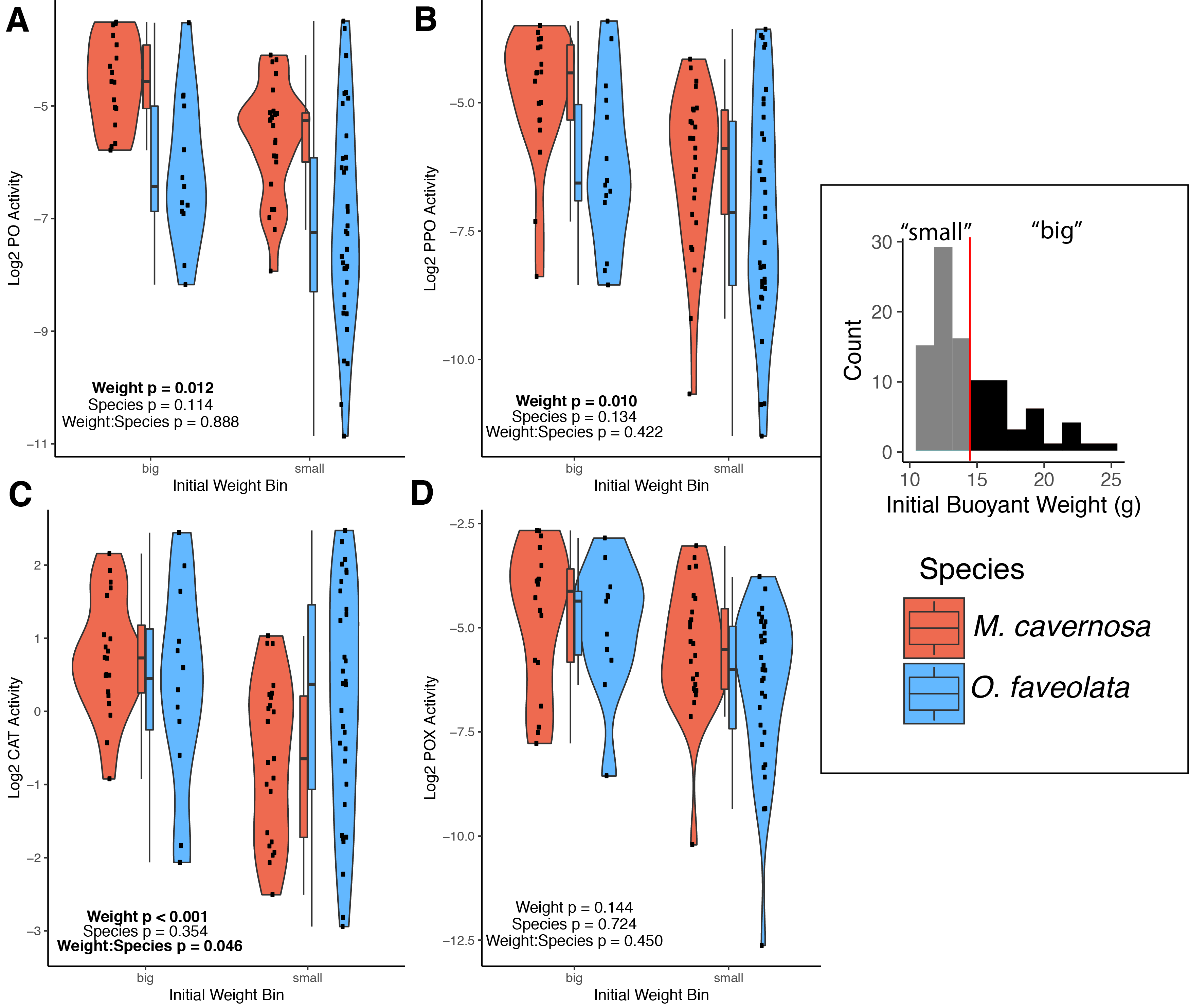

Supplement: Supplemental Information 2 — The histogram in the inset key depicts the range of initial weight values with a vertical red line indicating the cut-off point to bin fragments as “small” or “big”. Each point within the violin plots represents an individual coral fragment. Box plots depict the median (thick horizontal line) and interquartile range (box). P-values represent results from the MCMC model testing the individual effects of and interaction between weight and species. Significant terms are indicated in bold. [file peerj-10-13158-s002.png]

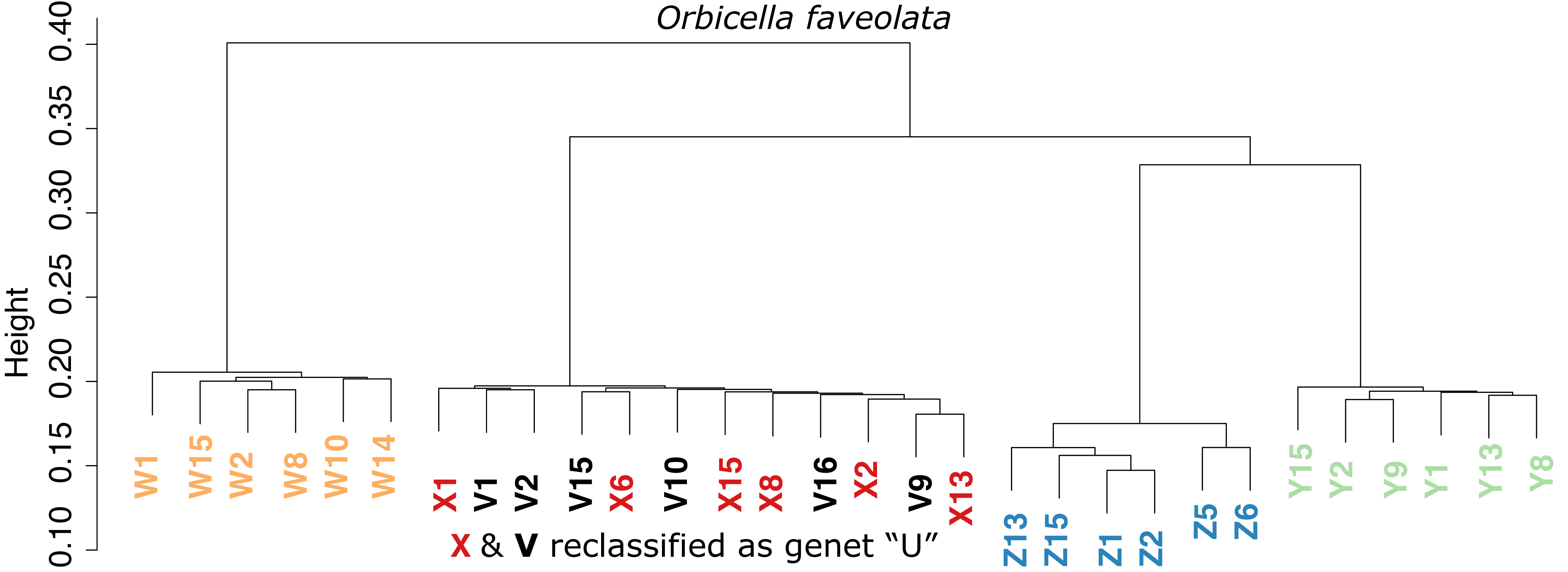

Supplement: Supplemental Information 3 — The color-coded sample name includes a letter representing a presumed genetic identity and a number indicating a replicate of that clone. Distances <0.2 are presumed clones based on clustering of replicate fragments within each genet. Former genets “X” and “V” are subsequently reidentified as genet “U”. [file peerj-10-13158-s003.png]

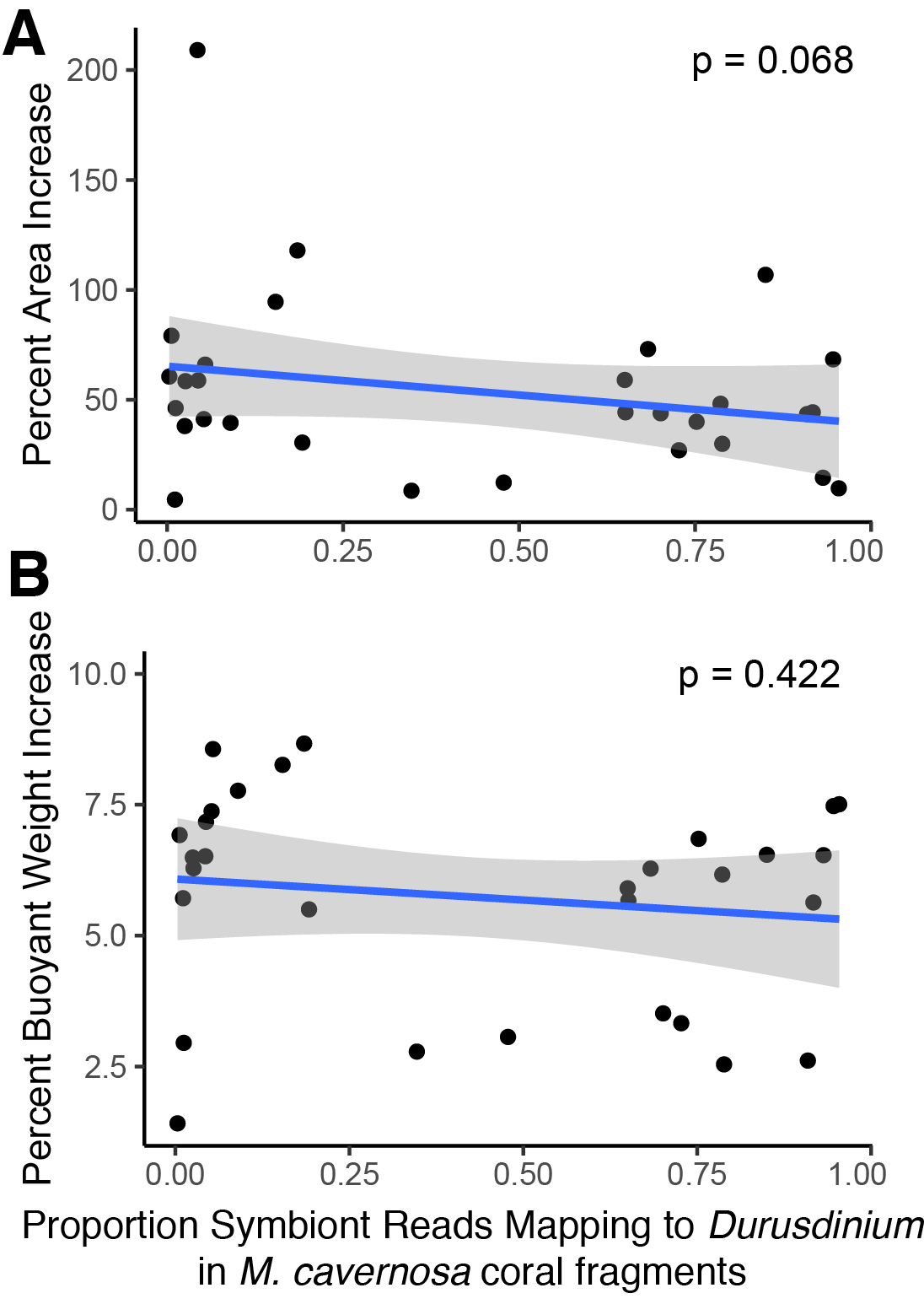

Supplement: Supplemental Information 4 [file peerj-10-13158-s004.png]

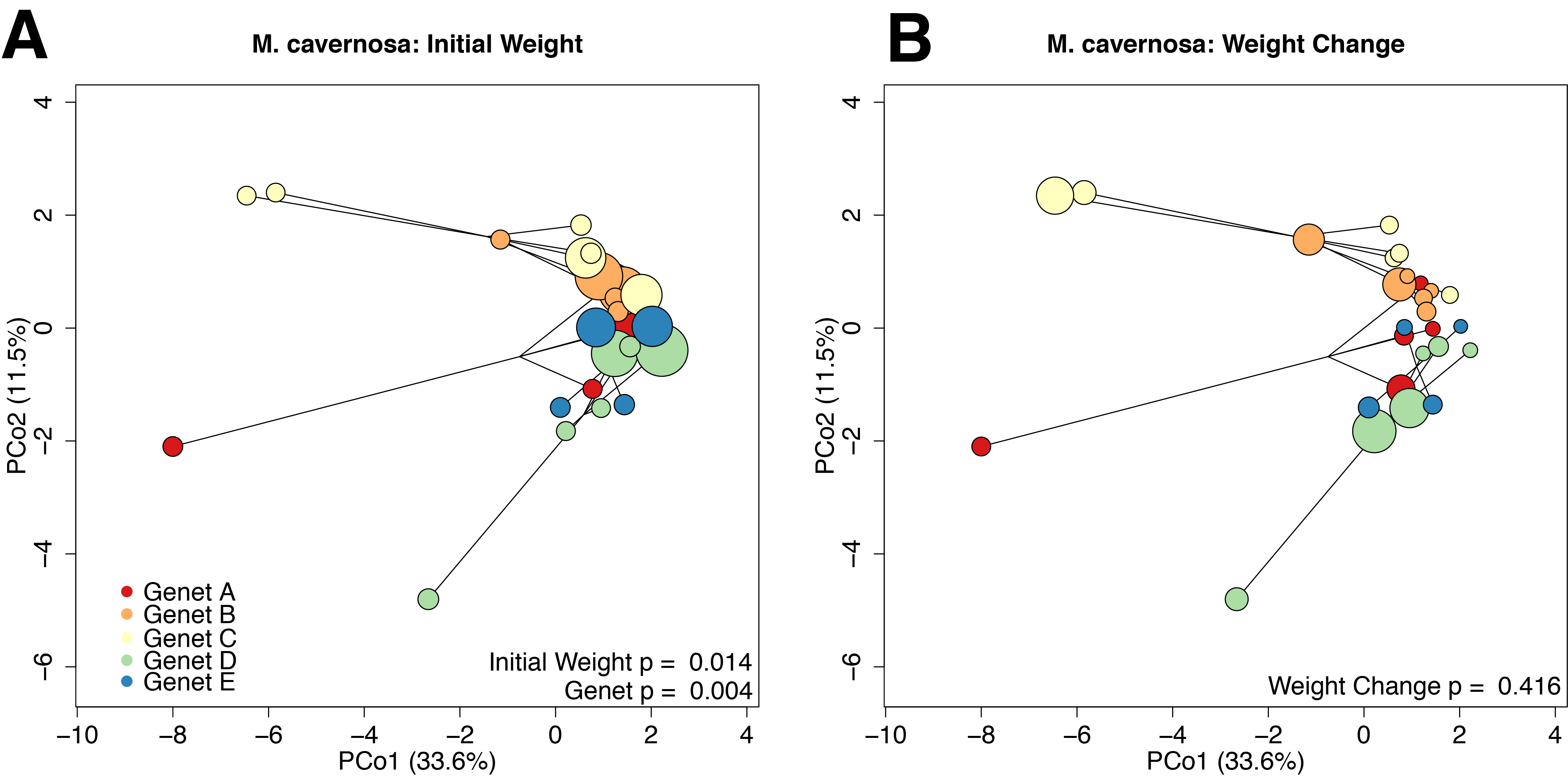

Supplement: Supplemental Information 5 — Individual points represent independent gene expression libraries. Colors correspond to genets according to the inset legend. Point size reflects the relative initial weight of the fragment (A) or relative weight change over the 4 month recovery period (B). P-values were generated by permutational multivariate analysis of variance using distance matrices. [file peerj-10-13158-s005.png]

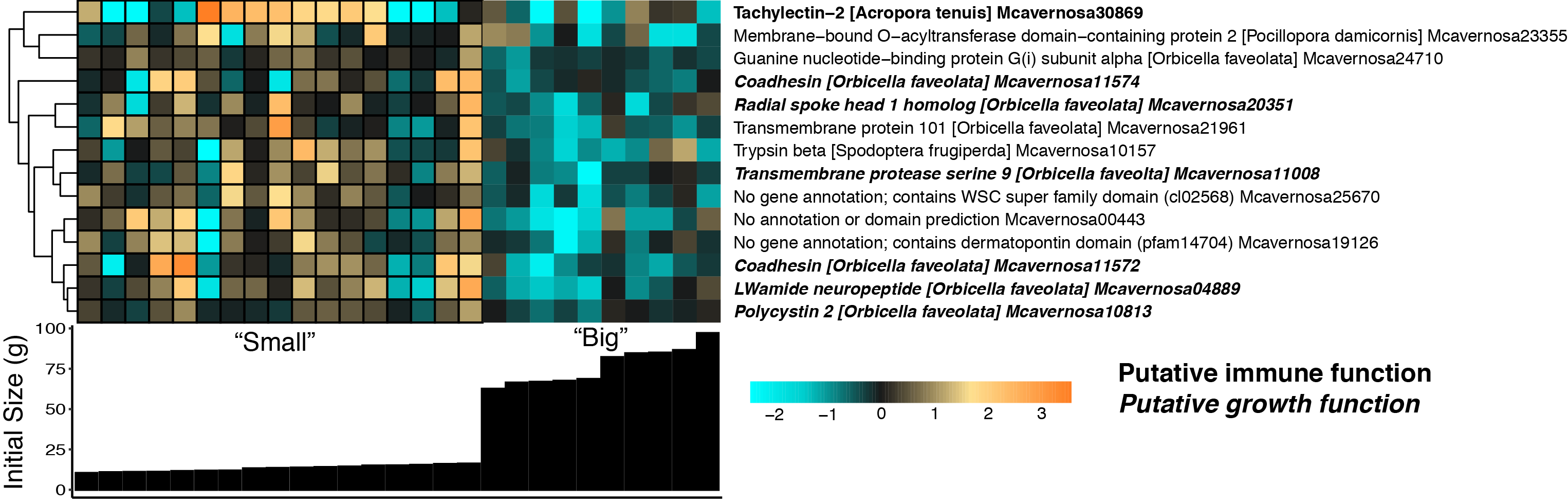

Supplement: Supplemental Information 6 — Rows are genes and columns are samples. The color scale indicates log2-fold change relative to the mean expression of each gene across all samples. Genes are hierarchically clustered based on Pearson’s correlations of expression across samples. Bar graphs below each column represent the initial buoyant weight (g). Genes with a putative immune function are highlighted in bold; genes with a putative growth function are bolded and italicized. [file peerj-10-13158-s006.png]

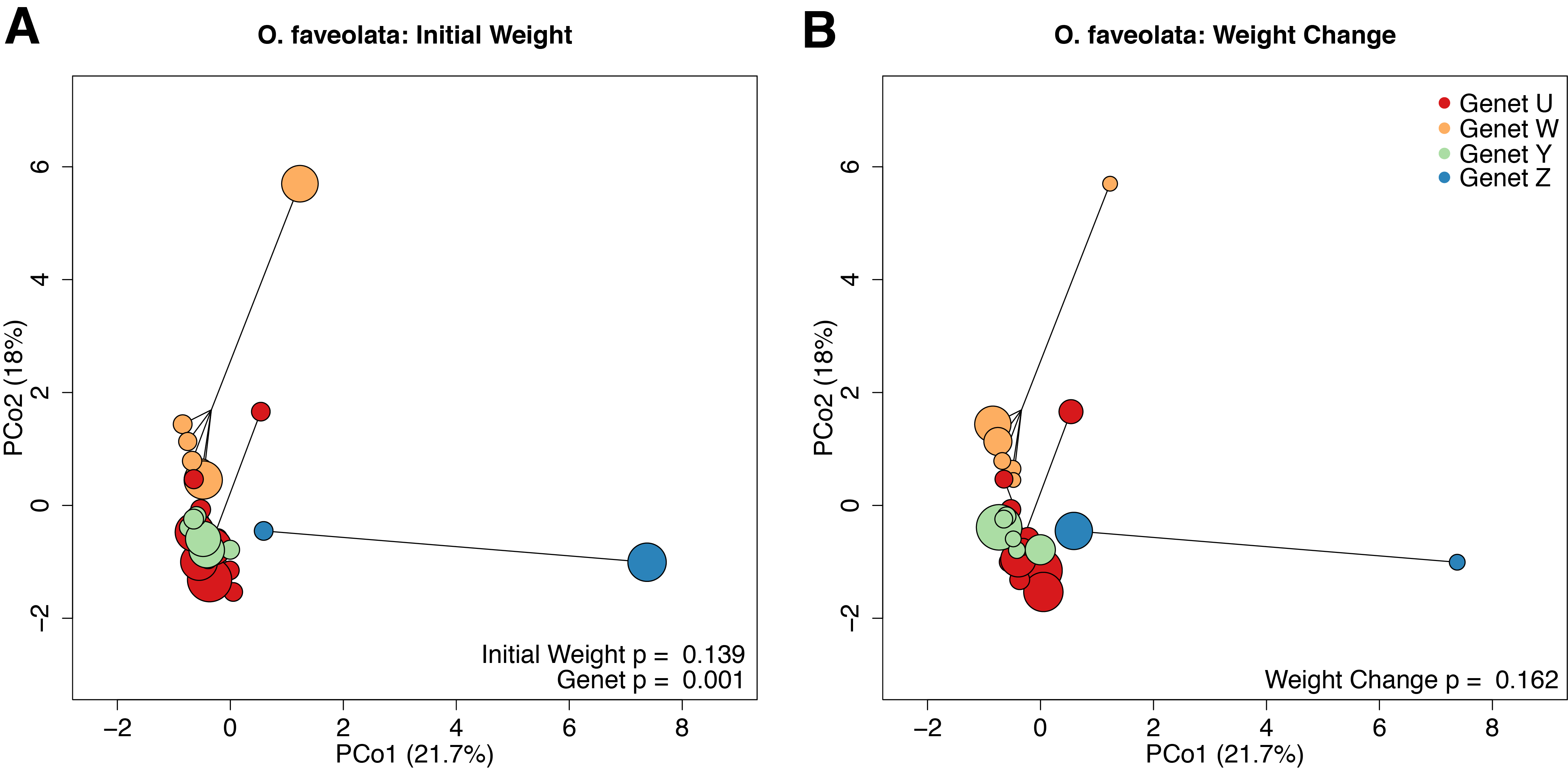

Supplement: Supplemental Information 7 — Individual points represent independent gene expression libraries. Colors correspond to genets according to the inset legend. Point size reflects the relative initial weight of the fragment (A) or relative weight change over the 4-month recovery period (B). P-values were generated by permutational multivariate analysis of variance using distance matrices. [file peerj-10-13158-s007.png]

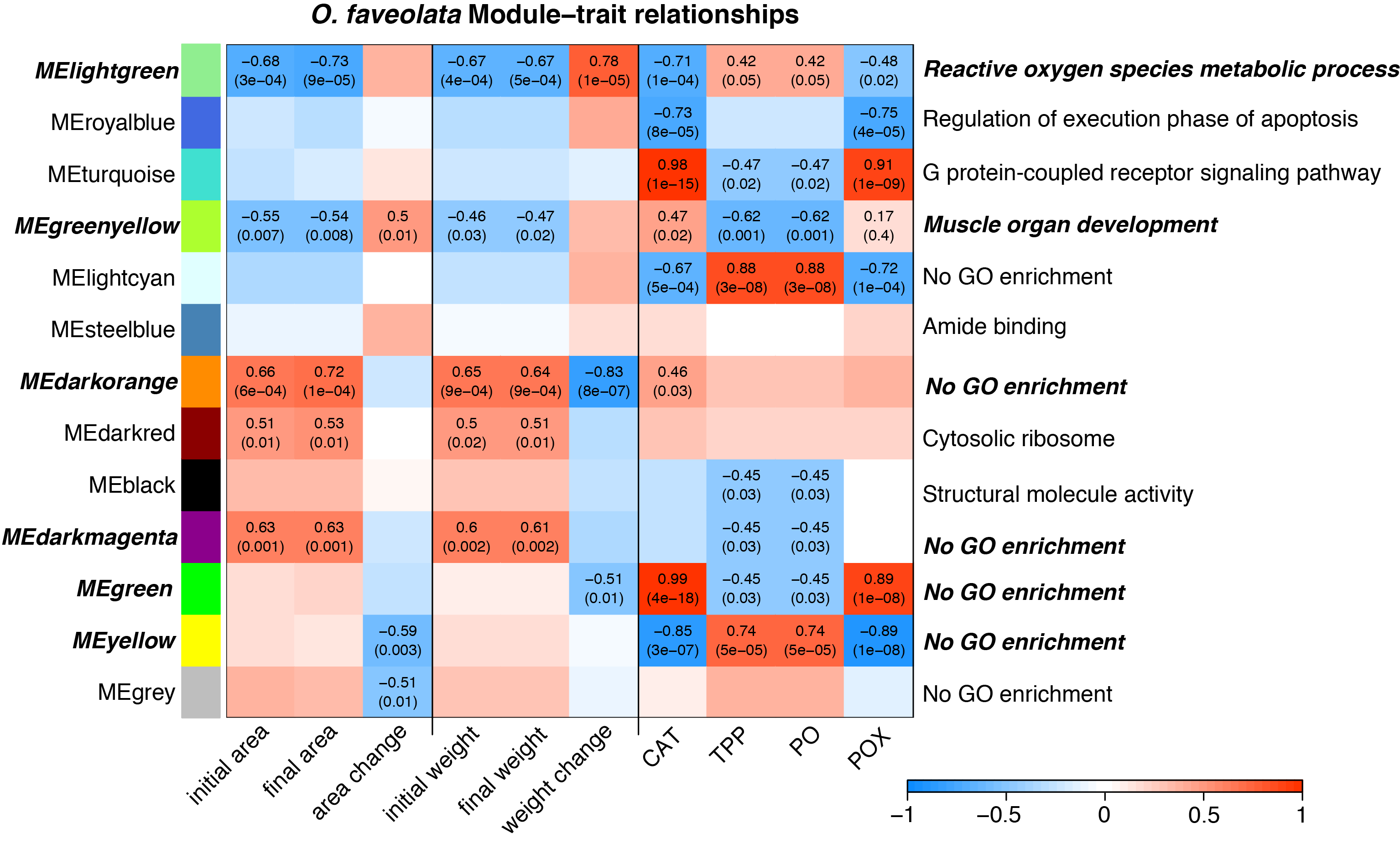

Supplement: Supplemental Information 8 — The strength of the correlations between traits (terms indicated on along the x-axis) and gene coexpression modules (colored boxes along y-axis) are indicated by the intensity of color. Values within each cell indicate Pearson’s correlation between the module eigengene and the trait and the p-value according to the correlation test for only significant correlations (p < 0.05). Terms along the right indicate a representative top enriched GO category for the module, if any. Modules in bold italic represent potential tradeoffs with opposite associations between growth and immune parameters. [file peerj-10-13158-s008.png]

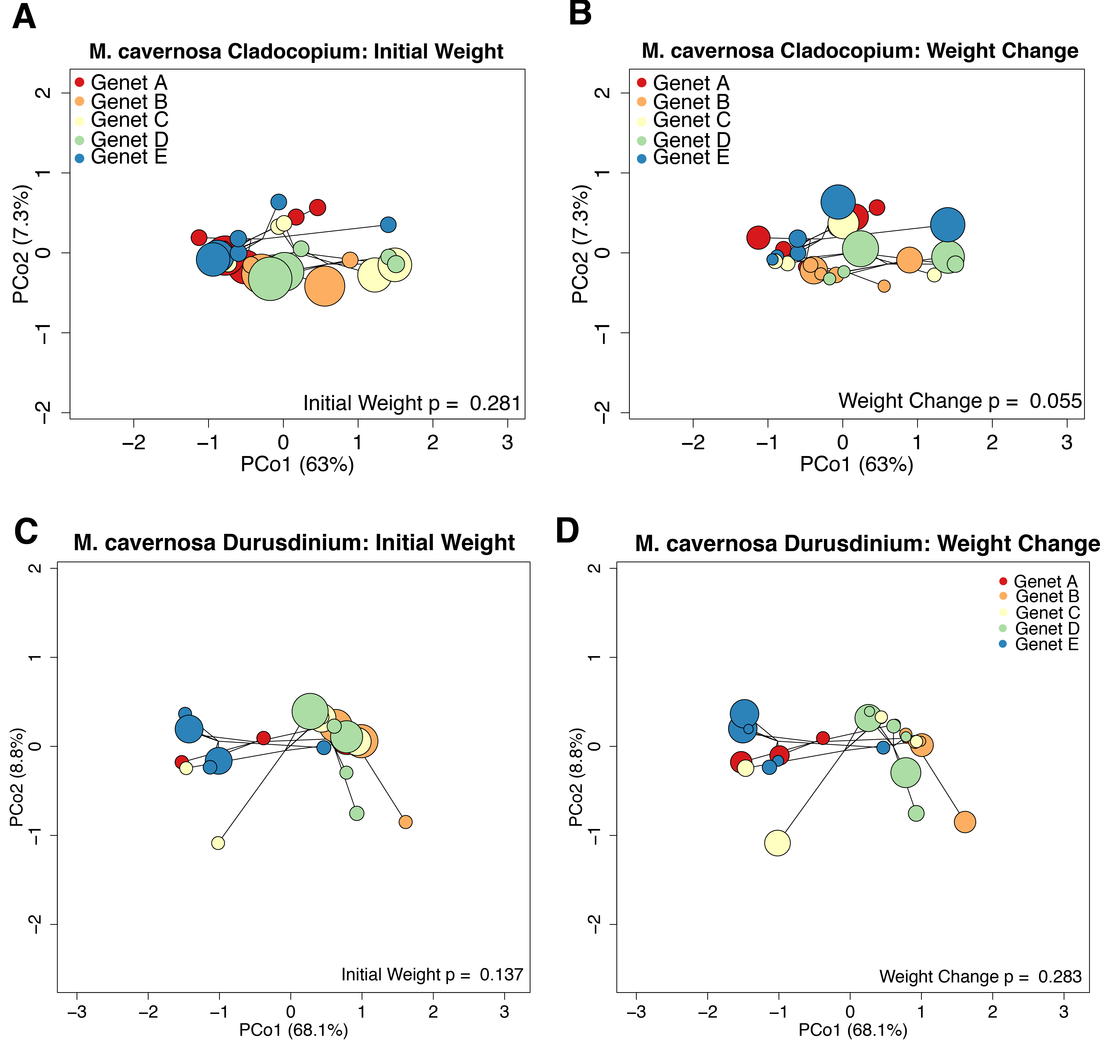

Supplement: Supplemental Information 9 — Individual points represent independent gene expression libraries. Colors correspond to genets according to the inset legend. Point size reflects the relative initial weight of the fragment (A and C) or relative weight change over the 4-month recovery period (B and D). P-values were generated by permutational multivariate analysis of variance using distance matrices. [file peerj-10-13158-s009.png]

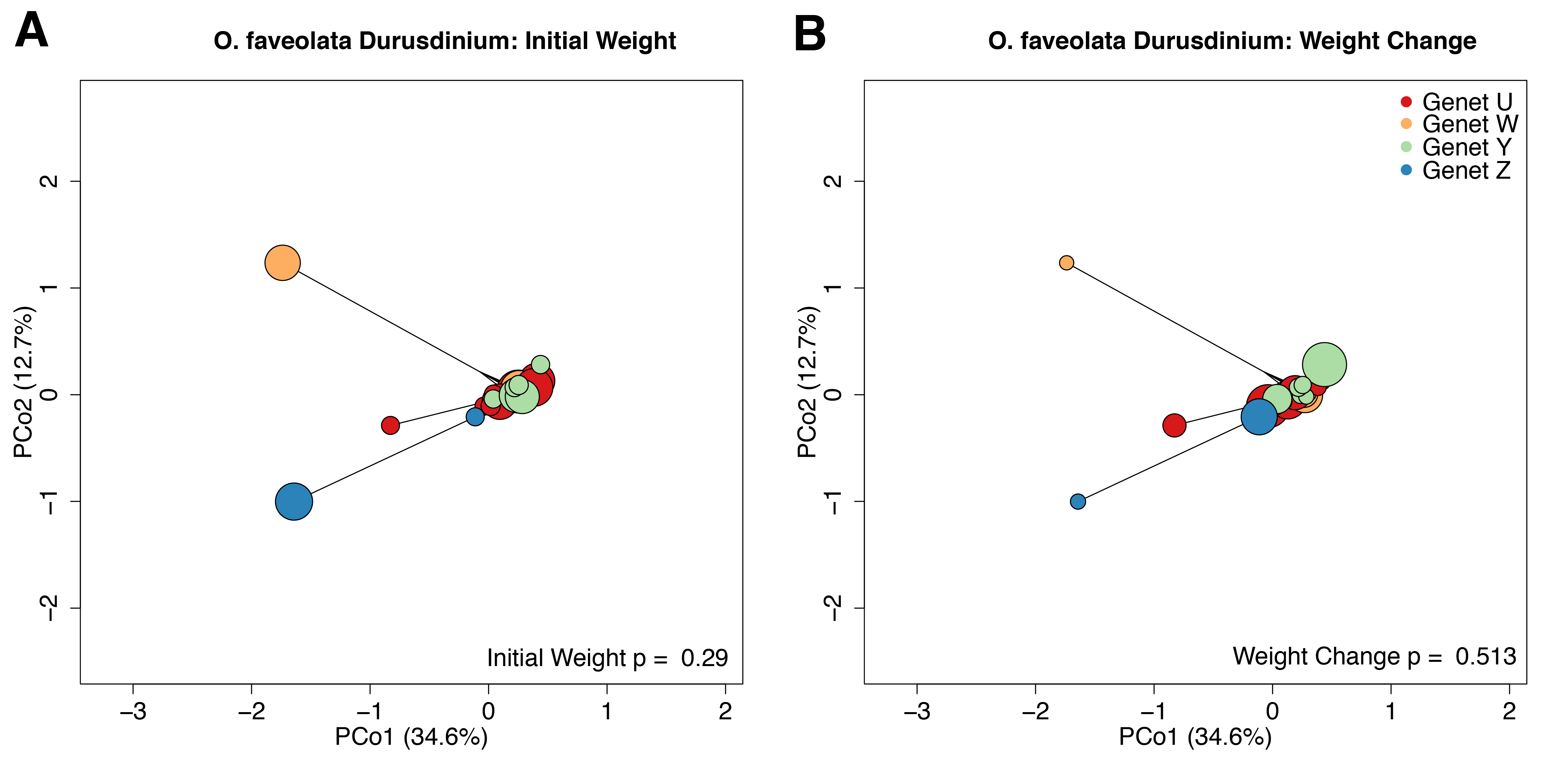

Supplement: Supplemental Information 10 — Individual points represent independent gene expression libraries. Colors correspond to genets according to the inset legend. Point size reflects the relative initial weight of the fragment (A) or relative weight change over the 4-month recovery period (B). P-values were generated by permutational multivariate analysis of variance using distance matrices. [file peerj-10-13158-s010.png]
